# STATE VARIATION IN NURSING HOME CIVIL MONEY PENALTY ENFORCEMENT ACTIONS FOR QUALITY DEFICIENCIES

**DOI:** 10.1093/geroni/igz038.567

**Published:** 2019-11-08

**Authors:** Denise L Gammonley, Xiaochuan (Sharon) Wang, Felicia Bender

**Affiliations:** University of Central Florida, Orlando, Florida, United States

## Abstract

States vary in their overall rates of nursing home deficiency citations as well as deficiencies for actual harm or jeopardy (Harrington et al., 2018). Civil Money Penalty (CMP) fines collected by the Centers for Medicare and Medicaid Services (CMS) are one enforcement action imposed to promote nursing home compliance with regulations. Collected CMP funds are redistributed to states for the sole purpose of improving nursing home resident care and quality of life through reinvestment in quality improvement projects. Using CASPER data available for US skilled nursing homes in 2015 and 2016 through the CMS QCOR database we examined the distribution of quality of care (QOC) and quality of life (QOL) deficiencies and CMP enforcement action across states. Guided by the systems framework for evaluating nursing home quality (Unruh & Wan, 2004) we further explored how contextual factors such as state spending for nursing home care, structural characteristics of facilities in states, and inadequate care processes indicated by deficiencies contribute to CMP enforcement actions and fines. Findings indicate that 27% of enforcement actions resulting in a CMP between 2015 and 2016 were imposed for a QOL deficiency while 61.7% represented QOC deficiencies. QOL deficiencies represented only 8% of the highest severity deficiency category but 81.7% of enforcement actions for QOC were for those causing immediate harm or jeopardy. QOC deficiencies are a focus of enforcement actions as they represent critical care processes influencing resident basic needs for hydration, ambulation, skin integrity and care for other special physical and behavioral needs.

